# Methylome Analysis in Nonfunctioning and GH-Secreting Pituitary Adenomas

**DOI:** 10.3389/fendo.2022.841118

**Published:** 2022-03-30

**Authors:** Giuseppe Giuffrida, Valeria D’Argenio, Francesco Ferraù, Vito Alessandro Lasorsa, Francesca Polito, Federica Aliquò, Marta Ragonese, Oana Ruxandra Cotta, Ylenia Alessi, Rosaria Oteri, Federica Di Maggio, Alessio Asmundo, Petronilla Daniela Romeo, Federica Spagnolo, Lucio Pastore, Filippo Flavio Angileri, Mario Capasso, Salvatore Cannavò, M’Hammed Aguennouz

**Affiliations:** ^1^ Department of Human Pathology DETEV, University of Messina, Messina, Italy; ^2^ Department of Human Sciences and Quality of Life Promotion, San Raffaele Open University, Rome, Italy; ^3^ CEINGE-Biotecnologie Avanzate, Naples, Italy; ^4^ Endocrine Unit, “Gaetano Martino” University Hospital, Messina, Italy; ^5^ Department of Molecular Medicine and Medical Biotechnologies, University of Naples Federico II, Naples, Italy; ^6^ Department of Clinical and Experimental Medicine, University of Messina, Messina, Italy; ^7^ Department of Biomedical and Dental Sciences, and Morpho-Functional Imaging, University of Messina, Messina, Italy

**Keywords:** GH-OMAs, methylation, pituitary adenomas, NFPAs, pituitary tumors

## Abstract

Pituitary adenomas (PAs), usually benign lesions, can sometimes present with “aggressive” features (rapid growth, local invasiveness, scarce response to conventional treatments). Despite the fact that a few genetic alterations have been associated to this clinical behavior, the role of epigenetic modifications, mainly methylation and miRNAs activity, is now opening new frontiers in this field. We evaluated the methylation profile of 21 PA (11 GH-omas, 10 nonfunctioning tumors—NFPAs) samples from TNS surgery and 5 normal pituitaries, collected at our neurosurgery between 2015 and 2017. DNA was extracted and sequenced, selecting 184,841 target regions. Moreover, methylation profiles were correlated with demographic, radiological, and clinicopathological features. NFPAs showed higher methylation levels vs. GH-omas, with 178 differentially methylated regions (DMRs) mainly consisting of noncoding and intronic sequences, and mostly localized in the open sea regions. We also found three hypermethylated genes (*C7orf50*, *GNG7*, and *BAHCC1*) involved in tumorigenesis processes and potentially influencing pituitary tumor pathophysiology. Among the clinicopathological features, only the maximum diameter resulted significantly higher in NFPAs. Our data provide further evidence of the complex epigenetic background of pituitary tumors. In line with the current literature, we confirmed a significant prevalence of hypermethylation in NFPAs vs. GH-omas, whose pathophysiological consequence is yet to be defined.

## Introduction

Pituitary adenomas (PAs) are distinguished by the presence of hormonal secretion and/or the expression of cell line-specific growth factors (1, 2). Although the presence of distant metastases is linked to the definition of pituitary carcinomas, even PAs can show an aggressive biological behavior, being characterized by local invasion, rapid proliferation, and scarce response to conventional treatments in up to 45% of cases (3, 4). In this context, there is a growing amount of data about PA (epi)genetic features predicting their behavior and/or their treatment response/relapse. In terms of genetics, for example, germinal mutations of the AIP (aryl hydrocarbon receptor-interacting protein) gene are associated to the development of familial isolated pituitary adenomas (FIPA), with early onset, higher aggressiveness, and resistance to somatostatin analogs (SSAs) (5). Similarly, the mutations involving the MEN1 oncosuppressor, linked to the homonymous syndrome, are associated with PAs in 15–50% of affected patients and a higher frequency of macroadenomas, that in 1/3 of cases are more invasive than non-MEN1 tumors (6). On the other hand, there is some evidence about somatic changes in sporadic pituitary tumors. These mutations can consist of sequence changes, qualitative alterations of chromosomes, or modification in their copy numbers, but they are often aspecific and infrequent, suggesting an additional oncogenic contribution from nonmutational factors (7, 8). Epigenetic modifications, which take place without altering the DNA sequences, comprehend both the alterations in mRNA transcription (nucleotides methylation, histones acetylation) and the different expression of long noncoding mRNAs (lncmRNAs) and, as also recently described by our group, microRNAs (miRNAs) (9). Methylation, that is, the apposition of methyl groups on DNA chains by specific enzymes—the DNA methyl-n-transferases (DMNTs)—is a physiological mechanism acting to silence specific genes in order to regulate their expression (8). Many DMNT isoforms are known, but DMNT1 and 3A are overexpressed in more aggressive PTs, with the DMNT1 more frequently found in macroadenomas (10). On the contrary, it seems that this DMNT hyperactivity would lead to hypomethylation of other DNA regions, which consequently result to being overtranscripted, as already observed in tumorigenesis processes (7). In such a context, the search for epigenetic changes can be crucial in order to identify potential predictors of clinical behavior and/or treatment response, as well as targets for tailored therapies (8). For example, in the case of GH-secreting PAs causing acromegaly, the presence of parameters predicting treatment response would be useful to avoid potentially inefficacious therapies that could have an impact on other conditions like glucose metabolism, or to guide drug dosing (11–14). Furthermore, even environmental factors, especially pollutants with endocrine disrupting activities, which have been increasingly demonstrated to have a role in PA pathophysiology, could have an impact on tumor epigenetic profile and molecular features, and consequently on their biological behavior (15–18).

This study aimed to assess the methylation status, as compared to normal pituitary tissues, of nonfunctioning pituitary adenomas (NFPAs) and GH-omas, and to correlate the methylation status of NFPAs and GH-omas with their epidemiological and clinicopathological features.

## Materials and Methods

### Tumor Sample Collection and DNA Extraction

Twenty-one PA samples (11 GH-omas, 10 NFPAs) were collected by the Neurosurgery Unit of Messina University Hospital between 2015 and 2017. All patients gave their written informed consent to the study. Demographic information, including sex, age, and clinical data, of the enrolled patients are summarized in **Table 1**. Five nontumor pituitary tissue samples were collected through an autopsy of subjects who died due to non-endocrine causes. The research protocol was approved by the local ethics committee. For DNA methylation analyses (see below), genomic DNA was extracted from each collected tissue using the QIAamp DNA mini kit (Qiagen), according to the manufacturer’s instructions.

**Table 1:** Demographic, radiological, and clinicopathological features of the studied cohort of patients.

ID	Sex	h.r. areas	Age at diagnosis	Micro/macro-adenoma	dmax mm	Cavernous s. invasion*	Ki-67%	p53%	TNS surgeries	*AIP* mutation
**GH1**	F		27	Macro	22	No	2	0	1	
**GH2**	F	Yes	76	Macro	15	No	1	0	1	
**GH3**	F		46	Macro	10	No	2	<1	1	
**GH4**	M		NA	Macro	NA	No	NA	NA	1	
**GH5**	M		57	Macro	22.5	No	1		1	
**GH7**	F		48	Macro	11	No	1	2	1	
**GH8**	F		28	Macro	18		5	2	1	
**GH9**	F		22	Micro	5	No	<1		1	Yes
**GH10**	F		35	Macro	12	No	<1	0	1	
**GH11**	M	Yes	63	Macro	43	Yes	5	0	2	
**GH12**	M		57	Macro	11	Yes	<1	0	1	
**total**	4M, 7F					2/11				
**median**			47		12		2		1	
**SD**			17.69		11.13		1.81		0.31	
**NFPA1**	F		46	Macro	30	Yes	3	1	1	
**NFPA5**	F		46	Macro	22	No	<1		1	
**NFPA6**	M		42	Macro	30	No	NA	NA	1	
**NFPA8**	F		NA	Macro	NA	No	NA	NA	1	
**NFPA11**	F	Yes	55	Macro	15	No	2		1	
**NFPA13**	M		68	Macro	30	No	2	<1	1	
**NFPA14**	F		70	Macro	25	No	<1		1	
**NFPA16**	M		72	Macro	23	Yes	<1		1	
**NFPA18**	M		40	Macro	34	Yes	1		2	
**NFPA19**	F		36	Macro	30	Yes	1	3	3	
**total**	4M, 6F					4/10				
**median**			46		30		2		1	
**SD**			13.94		5.60		0.83		0.70	
P value**	0.78		0.36		***0.02***	0.53	0.48		0.35	

NFPA, nonfunctioning pituitary adenoma; AIP, arhyl hydrocarbon receptor interacting protein (gene); cavernous s., cavernous sinus; h.r. areas, areas classified at high risk for health (highly polluted) by the Italian Government; NA, not available; SD, standard deviation; TNS, trans-nose sphenoidal.

*Cavernous sinus invasion defined by mean of 1.5 T MRI (magnetic resonance imaging) studies.

**P-value set at <0.05. The P-value 0.02 (in bold italic) indicates statistical significance.

### Whole-Genome DNA Methylation Sequencing

The whole-genome DNA methylation profiling of 11 GH-omas, 10 NFPAs, and 5 normal pituitaries was carried out with the TruSeq Methyl Capture EPIC library preparation protocol followed by next-generation sequencing (NGS) (Illumina). Genomic DNAs underwent picogreen quantification on the Qubit fluorimetric system (dsDNA HS assay, Life Technologies) in order to obtain 1,000 ng of DNA/sample for subsequent library preparation. Libraries were carried out following the manufacturer’s instructions. In detail, 1,000 ng of each sample was sonicated (Covaris M220 System) to obtain small DNA fragments (average size 150–200 bp) as assessed by the Tape Station quality check system (High Sensitivity D1000, Agilent). After end repair and adapter ligation, these DNA fragments were enriched by hybridization with specific capture probes. The enriched fragments were bisulfite converted and amplified. These obtained libraries were checked for quantity (Qubit, dsDNA HS assay, Life Technologies) and quality (Tape Station, High Sensitivity D1000, Agilent) before sequencing. NGS was carried out on the Illumina HiSeq1500 System. Up to 12 different DNA libraries, each univocally identified by a specific barcode or index, were pooled in equimolar amounts and sequenced in 4 different lanes in order to avoid analytical biases.

### Methylation Sequencing Data Analysis

A multistep bioinformatic pipeline was used to analyze the obtained sequencing data. First, sequencing reads quality check was carried out using the FASTQC software. For the sequence alignment and downstream quantification steps, we used the “QuasR” (version 1.22.1), an R-Bioconductor package installed on R (version 3.5.0) (19). The QuasR package integrates the functionality of several R packages for genomic intervals and alignment files manipulation and external software [e.g., Bowtie (20)] for the real sequence alignment. Sequence mapping was carried out using a BS pre-processed reference genome version (version GRCh37/hg19) that was generated exploiting the “QuasR” functions. The tool was run with default parameters. PCR duplicated reads were removed during the alignment. Subsequently, to quantify methylated and unmethylated cytosines in each sample, we used the function qMeth of the QuasR package. We considered a total of 437,792 genomic regions (mean length of 245 bp; from 2 to 8,131 bp) covered in the manifest file of the TruSeq Methyl Capture EPIC Library Prep kit (Illumina). In this step, the tool collapses the information of individual cytosines by query region. Finally, the methylation fraction of each target region for each sample was obtained as the ratio between methylated reads and the total number of aligned reads and ranged between 0 (un-methylated) and 1 (totally methylated). The tools were run with default parameters.

### Differential Methylation Analysis

For the differential methylation analysis, we considered GH-oma and NFPAs samples. To improve the consistency of the results, we kept all the target regions (n=184,841) that were covered in all the GH-oma and NFPAs samples and calculated the methylation fold enrichment (as Log2) between NFPAs and GH-omas mean methylation. Statistical significance was calculated with t-test, and P-values were corrected for multiple testing with the Bonferroni method. Significant results were considered if the Bonferroni adjusted P value was less than 0.05 and if the Log2 fold change was above or below 0.5. Functional annotation, to get distances from the nearest genes and other genomic information, of differentially methylated regions was performed with the ANNOVAR software.

The statistical evaluation of demographic and clinicopathological parameters was performed by means of t-test and chi-square test (with Yates’ correction), and significance was set at a P value less than 0.05.

## Results

Sequencing reads quality evaluation returned good-quality paired-end reads of length between 35 and 101 bp. The percentage of reads with quality scores above 20 (Q20) and above 30 (Q30) was 98.91 and 94.42, respectively (**Supplementary Figure 1A**). The mean base quality was 36.20 (**Supplementary Figure 1B**). The overall mapping rate ranged between 71.3% and 78.95%, as reported in **Supplementary Figure 1C**.

**Figure 1:**
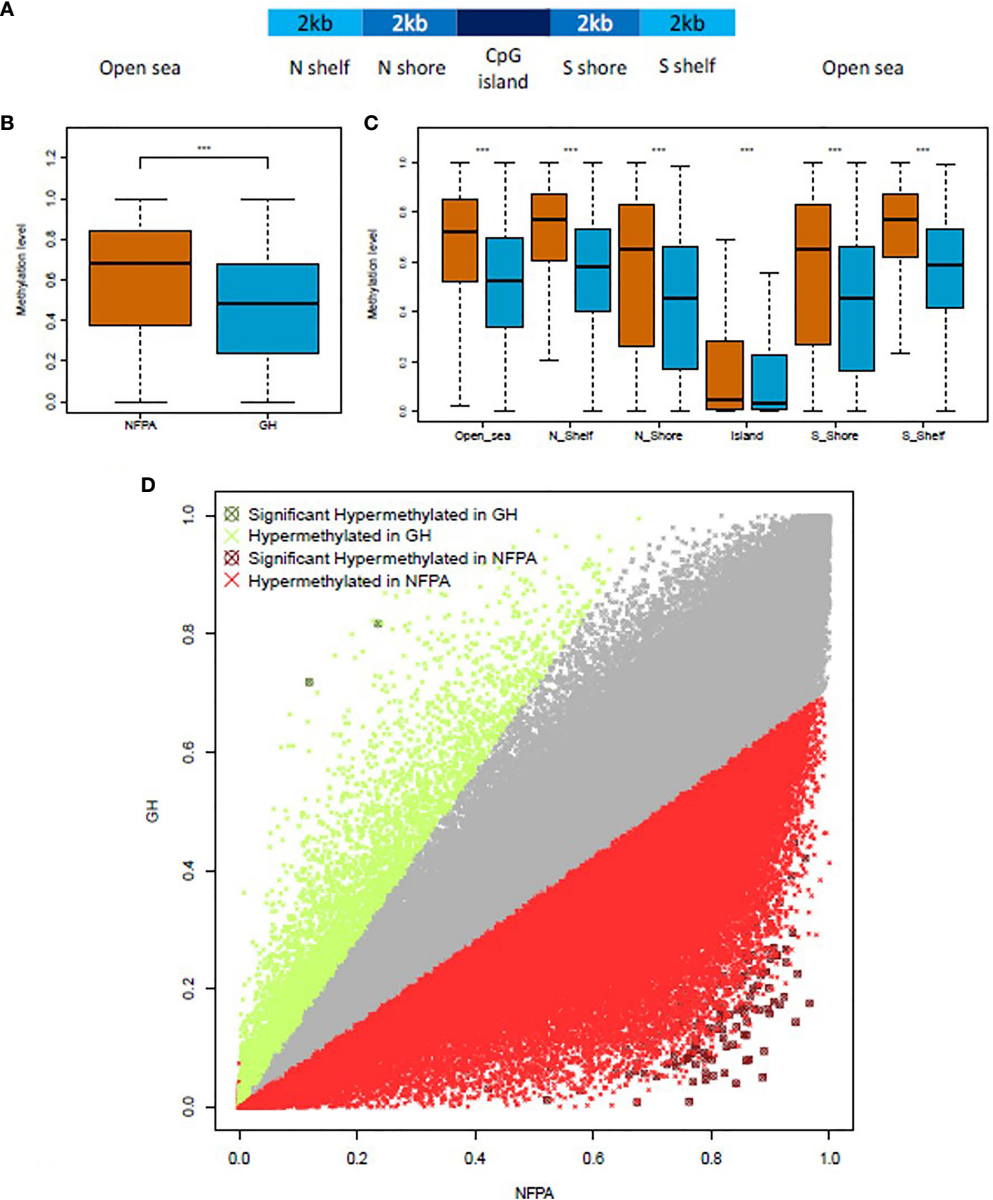
NFPAs are hypermethylated when compared to GH-secreting tumors. **(A)** Schematic representation of the CpG-related region annotation. **(B)** Boxplot showing the global level of methylation in nonfunctioning pituitary adenomas (NFPAs—brown) and GH-secreting (blue) tumors. **(C)** Methylation levels in CpG-related regions. **(D)** The scatterplot compares the methylation levels of 184,841 regions in NFPAs (x-axis) and GH-omas (y-axis). Data points in gray did not pass the Log_2_ fold change cutoffs. Points in red or green passed the Log_2_ fold change cutoffs. Points in dark red or dark green were significantly hypermethylated in NFPAs or GH, respectively. Mann–Whitney test was used in **(B, C)** t-test was used in **(D)**. P < 0.0001 (***).

The methylation quantification step was performed by restricting the analysis to the genomic regions covered in the TruSeq Methyl Capture EPIC manifest file provided by Illumina. A total of 437,792 regions with a mean length of 245 bp (from 2 to 8,131 bp), being equivalent to 301,525 non-CpGs, 61,703 CpG islands, 18,707 N_shelf, 19,038 N_shore, 19,201 S_shelf, and 17,618 S_shore regions, were analyzed. The general positioning of these sequences in the human genome is summarized in **Figure 1A**.

For the differential methylation analysis, we compared GH-oma and NFPAs samples. To improve the consistency of the results, we kept all the target regions (n=184,841) that were covered in all the GH-oma and NFPAs samples. As reported in **Figure 1B** and **Supplementary Figure 1D**, globally, NFPAs showed higher methylation levels (median=0.68) compared to GH-secreting pituitary tumors (median=0.48) (P<2.2x10^-16^; Mann–Whitney test). Moreover, we evaluated the methylation levels of CpG-related regions and found that NFPAs were hypermethylated, as compared to GH-secreting pituitary tumors. In particular, we found hypermethylation in Open sea (median=0.7210 vs median=0.5236, respectively); in N Shelfs (median=0.7707 vs median=0.5786, respectively); in N Shores (median=0.6534 vs median=0.4517, respectively); in Islands (median=0.04584 vs median=0.030993, respectively); in S Shores (median=0.6526 vs median=0.4569, respectively), and in S Shelfs (median=0.7748 vs median=0.5855, respectively) (**Figure 1C**; P<2.2x10^-16^; Mann–Whitney test).

Next, we calculated the methylation fold enrichment (as Log2) between NFPA and GH-oma samples to identify the differentially methylated regions (DMRs).

NFPAs showed a distinct methylation profile as compared to GH-omas. In particular, we obtained 178 target regions that were differentially methylated (corrected P-value ≤0.05; Log2 Fold Change ± 0.5) between the two tumor types (**Figure 1D** and **Supplementary Table 1**). Of note, only two regions resulted significantly hypermethylated in GH-omas compared to NFPAs (**Figure 1D**).

We classified as H-DMRs (high differentially methylated regions) those regions having Log2 FC above 2 or below -2. This category counted a total of 111 DMRs (62.36%), one of which was hypomethylated in NFPAs as compared to GH-oma. DMRs with Log2 FC values between 2 and -2 were deemed as L-DMRs (low differentially methylated regions). This class included a total of 67 DMRs (37.64%), one of which was hypermethylated in NFPAs as compared to GH-oma.

**Figure 2:**
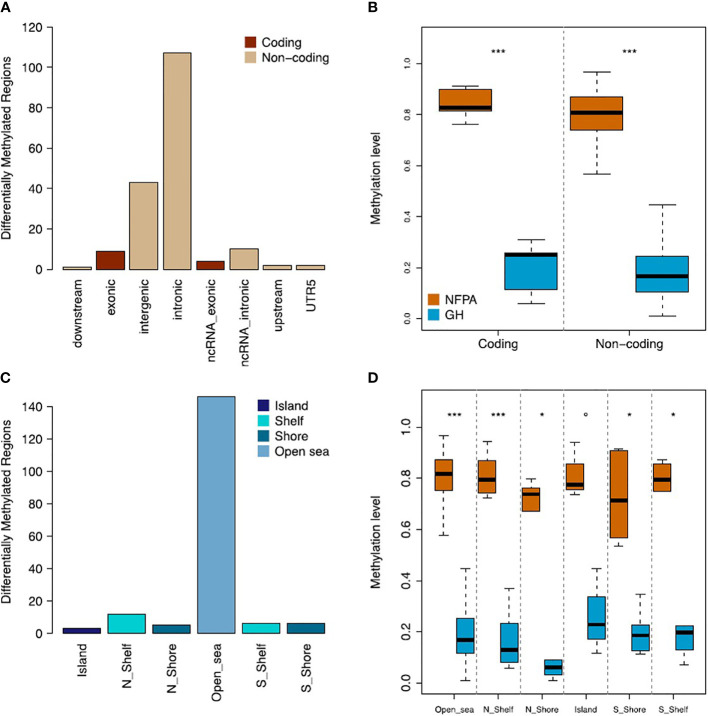
NFPAs and GH tumors are differentially methylated. **(A)** Barplot showing the gene annotation of the 178 DMRs. **(B)** Boxplot reporting the comparison of methylation levels of coding and noncoding regions between nonfunctioning pituitary adenomas (NFPAs) and GH tumors. **(C)** Barplot showing the CpG-related annotation of the 178 DMRs. **(D)** Boxplot reporting the comparison of methylation levels of CpG-related regions between NFPAs and GH tumors. Mann–Whitney test was used in **(B, D)** P = 0.1 (°), P < 0.01 (*), P < 0.0001 (***).

Subsequently, we functionally annotated the list of DMRs and found that the majority of them mapped in noncoding regions (92.7%), of which 64.85% were intronic sequences (**Figure 2A**). Moreover, we found that DMRs within the coding regions were significantly hypermethylated in NFPAs (median beta value=0.83 and 0.25 for NFPAs and GH-oma, respectively; P = 1.92x10^-07^). Accordingly, also noncoding sequences were significantly hypermethylated in NFPAs (median beta value=0.81 and 0.16 for NFPAs and GH-oma, respectively; P = 4.38x10^-53^) (**Figure 2B**, and **Supplementary Table 1**).

The CpG-centric annotation of DMRs (see **Figure 1A**) highlighted that the large majority of DMRs were annotated as Open sea (82.02%) (**Figure 2C**). These DMRs were significantly hypermethylated in NFPAs (median beta value=0.82 and 0.17 for NFPAs and GH-oma, respectively; P = 4.60x10^-47^). Overall, as reported in **Figures 2B, D**, we observed generalized hypermethylation in NFPAs as compared to GH-oma (P < 4.38x10^-53^).

**Figure 3:**
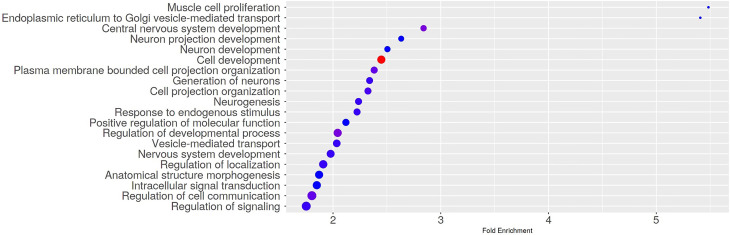
Gene Ontology Biological Process Enrichment results. The dotplot shows the top 20 significantly enriched GO terms. The Gene Ontology Biological Process Enrichment Analysis was run using the web app ShinyGO (v0.741) with the FDR cutoff set to 0.05.

To assess if the DMR-related genes were involved in specific pathways, we conducted a Gene Ontology Biological Process Enrichment Analysis using the web app ShinyGO (v0.741) (PMID: 31882993) and set the FDR cutoff to 0.05. Of note, among the significantly enriched GO terms, we found biological processes related to cell and neuron development (**Figure 3** and **Supplementary Table 2**).

With regard to the correlation between methylation profile and demographic (including the degree of pollution of the residence area) or clinicopathological features of pituitary tumors, no statistically significant differences were observed between GH-omas and NFPAs, except for the maximum tumor diameter (**Table 1**), which resulted significantly higher in the latter group (median ± SD: 30 ± 5.6 vs 12 ± 11.13 mm; P = 0.02). Of note, 4 out of 10 (40%) patients with NFPAs presented with a neuroradiologically documented invasion of cavernous sinus vs 2 out of 11 (18.2%) in the GH-oma group, but this difference was not statistically significant (**Table 1**).

Finally, we found three hypermethylated genes (*C7orf50*, *GNG7*, and *BAHCC1*), involved in tumorigenesis processes, whose role could be related to pituitary tumor pathophysiology.

## Discussion

The role of epigenetic modifications, especially methylation, has increased its importance in the genetic background of sporadic pituitary tumors in the last few years. In fact, only a few somatic mutations with significant effects are known, such as *GNAS* alterations (this gene codifies for the α stimulatory subunit of G proteins) in GH-omas or *USP8* mutations in ACTH-omas causing *EGFR* overexpression and promoting corticotroph cells growth and ACTH hypersecretion (21). Also, our group recently demonstrated a novel somatic deletion in exon 10 of the *AHR* (aryl hydrocarbon receptor) gene in patients affected by GH-omas, whose role could be related to an altered AHR/AIP pathway favoring tumorigenesis (22). On the other hand, in pituitary tumors, it has been observed that methylation is preferentially concentrated in the so-called CpG islands, sequences of about 500 bp strictly connected to promoter regions, leading to the silencing of genes often involved in cell cycle regulation (23). Of note, a lot of oncosuppressors can be found among these genes, as for the couple *CDKN2A/Rb1*, whose deregulation can cause altered apoptosis regulation (8). In fact, methylation of *CDNK2A* leads to a reduced expression of p16, which, in turn, determines pRb phosphorylation and cell cycle progression through the activation of E2F transcription factors (24). However, the hardest challenge in the assessment of pituitary tumor methylation profile is to find a consistent “signature,” potentially useful as biological/prognostic/therapeutic marker. As it emerges from many studies—also confirmed by our findings—NFPAs tend to present with a higher degree of methylation compared to GH-omas, although some invasive NFPAs can even be characterized by hypomethylation (7, 23, 25, 26). Besides, NFPAs more frequently harbor *CDKN2A/p16* alterations, inversely from what were observed in GH-secreting pituitary tumors, which often do not express pRb (8). *CDKN2A* methylation has been related to the pituitary tumor volume, grade, and patients’ age, with higher methylation levels in macroadenomas (8). Furthermore, p27 hypermethylation was found in ACTH-omas, while *EML2*, *HOXB1*, and *RHOD* epigenetic modifications have been reported in NFPAs, GH-omas, and PRL-omas, respectively (7, 8). Gu et al. demonstrated that methylation would lead to the downregulation of genes like *GALNT9*, *CDH1*, and *CDH13* (E-cadherin and H-cadherin, respectively), involved into cellular adhesion processes, and potentially linked to the development of invasiveness (26). The same study observed that DMRs were located not only in CpG islands but also in the gene body in 40% of the cases (26). Accordingly, in our study, only 3 DMRs were found in known CpG islands, while the remaining alterations were found in genome open sea regions. Other genome elements prone to methylation are lncRNAs, RNA fragments of about 200 nucleotides functionally similar to the respective coding RNAs (27). In this regard, the downregulation following the hypermethylation of *MEG3*, which interacts with p53 and acts as oncosuppressor, has been found in gonadotropinomas (8, 27, 28). Another lncRNA, called C5orf66-AS1, regulates several genes, including *PAQR7*, a progesterone receptor that causes a progesterone A-B receptor-independent reduction in GnRH, whose expression has been found to have a role in progression and invasion of null-cell pituitary adenomas (24, 29).

With regard to our findings, DMR analysis revealed a prevalence of methylation of noncoding sequences, including lncRNAs (**Figure 2A**). Most of the methylation profile alterations were localized in open-sea regions more than involving promoters, with an inverse trend if compared to the literature (**Figure 2C**). Anyway, the prevalence of hypermethylation in NFPAs vs GH-omas has been confirmed (**Figures 2B, D**). Interestingly, we found hypermethylation of 3 known CpG islands belonging to genes thought to have a role in tumorigenesis processes: *C7orf50*, *GNG7*, and *BAHCC1*.

*C7orf50* is a ubiquitarian gene whose product is implicated in the assembling of ribosomal RNA to the nucleus, even if part of its sequences could also originate some regulatory miRNAs. Its full function is still unknown, although some evidence suggests it could bind the Sp1 transcriptional factor, which has several regulatory functions (i.e., cell cycle, apoptosis, etc.) including an interaction with *AHR* favoring the ubiquitination and consequent degradation of the estrogenic receptor α (ERα) in murine breast and uterine cancer (30).

*GNG7* is a gene located on chromosome 19 codifying for the γ7 subunit of guanin-binding G proteins, which is involved in contact-mediated cell growth blockade and acts as an oncosuppressor (31), whose promoter methylation has been found in many cases of head/neck cancer and associated with higher tumor volume and lesser metastatic potential (31). Similarly, Xu et al. observed methylation-mediated, reduced expression of *GNG7* in renal clear cell carcinoma. In this case, methylation, not present in normal tissue, led to the impairment of the mTOR1 signaling pathway and was linked to a higher stadium/grade of the disease and a reduced overall survival (32).

*BAHCC1* is a chromatin transcriptional silencer, implied into cell replication and transcriptional regulation mechanisms. Amplifications and deletions of this gene would make it potentially part of aberrant cell regeneration processes linked to the development of liver cancer, according to still-not-well-known mechanisms, but possibly due to downstream alterations in the signaling pathways (33). However, there are few data about epigenetic modifications of *BAHCC1*, although an experimental study by Gitik et al. found an increase in its methylation in the dorsal hippocampus of mice treated with nicotine before adolescence, in an animal model correlating substance abuse in the age of adolescence (or alcohol exposure *in utero*) with addiction. These chromatin modifications were linked to the development of cognitive deficits in the adult age, otherwise preventable by the simultaneous administration of choline (34).

With regards to the correlation between methylation profile and clinicopathological features, the higher maximum diameter of NFPAs could hypothetically be linked to a higher proliferative potential in this subtype of pituitary tumors. The same could apply to the higher frequency of cavernous sinus invasion—although not statistically significant—in the NFPA group. These findings are in line with the study by Gu et al. in which hypermethylation altered the expression profile of cell adhesion proteins (26). Finally, no relationship was observed between the methylation status and the degree of pollution of the residence area of our patients, but the number of pituitary tumors evaluated in this study is very small. Furthermore, the hypermethylation of *C7orf50*, a gene interacting with *AHR*, should be investigated in larger cohorts of patients. In fact, better defining such an interaction could add new information to the complex role played by *AHR*, which along the years we demonstrated to significantly influence morphology, secretion, and therapeutic response in GH-omas (16–18, 35).

In conclusion, our data provide further evidence on the complexity of the epigenetic background of pituitary tumors. We found a significant prevalence of hypermethylation in NFPAs, as compared to GH-omas, whose pathophysiological consequence is yet to be defined. Further studies are needed to clarify the role and relevance of *C7orf50*, *GNG7*, and *BAHCC1* genes—which have been found to be methylated—in pituitary tumor biology, oncogenesis, and clinical expression.

## Data Availability Statement

The data presented in the study are available on the European Nucleotide Archive (ENA) repository. Accession nr: PRJEB50807.

## Ethics Statement

The studies involving human participants were reviewed and approved by the Ethics Committee of The Province of Messina. The patients/participants provided their written informed consent to participate in this study.

## Author Contributions

GG and FF wrote the paper and organized research data. GG, MR, OC, FS, and YA contributed to the gathering of clinical data. FP, FAl, PR, RO, and M'HA (Messina) and VD’A, FM, LP, VL, and MC (Naples) performed DNA sequencing and methylome analysis. FAn provided samples from patients by means of TNS surgery, while AA provided healthy pituitary samples from autopsies. FF, SC, M'HA, and MC conceived the entire study and revised the final paper version. All authors contributed to the article and approved the submitted version.

## Funding

This work was supported by the following grants of the Italian government: Ricerca Finalizzata 2013: “Role of environment-gene interaction in etiology and promotion of pituitary tumours” (code: RF-2013-02356201); Programma di Ricerca di Interesse Nazionale 2015: “Epidemiological determinants, molecular mechanisms and clinical criteria of treatment outcome and resistance in pituitary disease syndromes” (code: PRIN-2015-2015ZHKFTA); and Progetto Rilevante di Interesse Nazionale 2017: “Identification of new biomarkers and clinical determinants for management improvement of patients with pituitary tumor related syndromes” (code: PRIN 2017S55RXB).

## Conflict of Interest

The authors declare that the research was conducted in the absence of any commercial or financial relationships that could be construed as a potential conflict of interest.

## Publisher’s Note

All claims expressed in this article are solely those of the authors and do not necessarily represent those of their affiliated organizations, or those of the publisher, the editors and the reviewers. Any product that may be evaluated in this article, or claim that may be made by its manufacturer, is not guaranteed or endorsed by the publisher.

## Supplementary Material

Supplementary Figure 1 Evaluation of sequencing, mapping, and methylation analysis data. **(A)** Boxplot reporting the percentage of reads with quality scores above 20 (Q20) and above 30 (Q30). **(B)** Boxplot reporting the mean base quality of sequencing reads. **(C)** Summary of the mapping rates. **(D)** Boxplot of the global methylation level of the 437,792 regions analyzed.
