# Supplementary figures and images for: Methylome Analysis in Nonfunctioning and GH-Secreting Pituitary Adenomas
Source: Front Endocrinol (Lausanne). 2022 Mar 30;13:841118. doi: 10.3389/fendo.2022.841118 (PMC9007725; doi:10.3389/fendo.2022.841118)

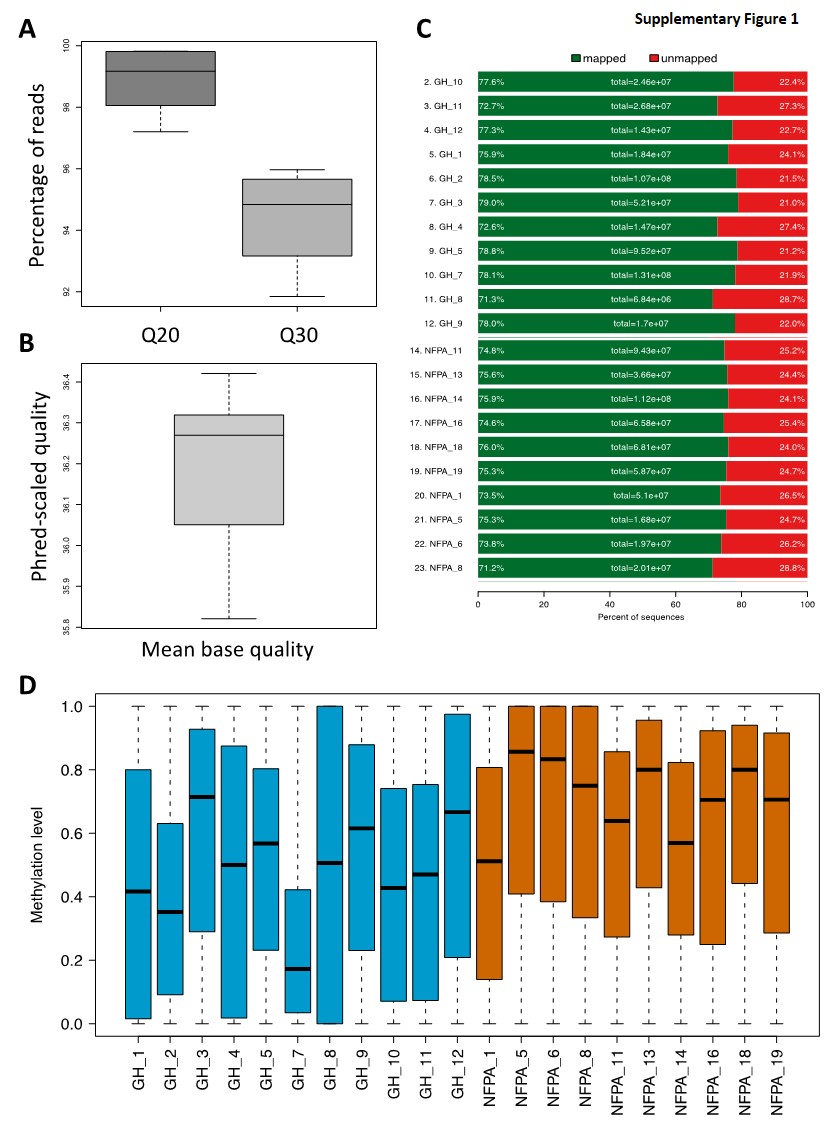

Supplement: Supplementary Figure 1 — Evaluation of sequencing, mapping, and methylation analysis data. (A) Boxplot reporting the percentage of reads with quality scores above 20 (Q20) and above 30 (Q30). (B) Boxplot reporting the mean base quality of sequencing reads. (C) Summary of the mapping rates. (D) Boxplot of the global methylation level of the 437,792 regions analyzed. [file Image_1.jpeg]
