# Supplementary material for: Methylome Analysis in Nonfunctioning and GH-Secreting Pituitary Adenomas
Source: Front Endocrinol (Lausanne). 2022 Mar 30;13:841118. doi: 10.3389/fendo.2022.841118 (PMC9007725; doi:10.3389/fendo.2022.841118)
Supplement: Supplementary file 2 [file Table_1.docx]

**Supplementary Table 1. Significant Differentially Methylated Regions annotation.**

| Chr: chromosome; CpG: CpG islands; Log2FC: Log2 Fold Change. |  |  |  |
| --- | --- | --- | --- |
| * Statistical significance was calculated with T-test and P-values were corrected for multiple testing with Bonferroni method. | | | |
| Significant results were considered if the Bonferroni adjusted P value was less than 0.05 and if the Log2 Fold Change was above or below 0.5. | | | |

| Chr | CpG name | CpG-related regions | Length | Gene function | Gene reference | cytoBand | Mean GHomas | Mean NFPAs | P value* | Log2FC* | DMR category |
| --- | --- | --- | --- | --- | --- | --- | --- | --- | --- | --- | --- |
| chr19 | . | . | 192 | intronic | INSR | 19p13.2 | 0,04995011 | 0,88694212 | 2,40E-06 | 4,1502802 | H-DMR |
| chr16 | chr16:27279886-27280243 | N_Shelf | 398 | intronic | NSMCE1 | 16p12.1 | 0,16990745 | 0,78893623 | 2,69E-06 | 2,215159579 | H-DMR |
| chr16 | . | . | 237 | intergenic | SMPD3(dist=50427),ZFP90(dist=40588) | 16q22.1 | 0,0897227 | 0,76442692 | 2,28E-05 | 3,090833644 | H-DMR |
| chr6 | chr6:168377416-168377724 | N_Shelf | 155 | intergenic | MLLT4(dist=1172),HGC6.3(dist=2577) | 6q27 | 0,14353506 | 0,94279283 | 3,45E-05 | 2,715537613 | H-DMR |
| chr15 | . | . | 251 | intronic | GPR176 | 15q14 | 0,0784716 | 0,85957218 | 3,58E-05 | 3,453376269 | H-DMR |
| chr5 | . | . | 207 | intronic | FCHO2 | 5q13.2 | 0,15751737 | 0,89876301 | 3,94E-05 | 2,512429821 | H-DMR |
| chr10 | . | . | 279 | intergenic | LDB3(dist=13778),BMPR1A(dist=6515) | 10q23.2 | 0,22503467 | 0,94591149 | 7,17E-05 | 2,071557908 | H-DMR |
| chr11 | . | . | 328 | intronic | RDX | 11q22.3 | 0,22841346 | 0,89287537 | 7,79E-05 | 1,966811145 | L-DMR |
| chr7 | . | . | 557 | intronic | HIPK2 | 7q34 | 0,14427171 | 0,86041752 | 0,00011859 | 2,57624847 | H-DMR |
| chr14 | . | . | 244 | ncRNA_intronic | RP11-58E21.3 | 14q21.3 | 0,04538391 | 0,79036285 | 0,00013153 | 4,122262323 | H-DMR |
| chr1 | . | . | 358 | intronic | CAPZB | 1p36.13 | 0,44640606 | 0,89841881 | 0,00014846 | 1,009031521 | L-DMR |
| chr1 | . | . | 284 | intronic | STMN1 | 1p36.11 | 0,1637391 | 0,88082633 | 0,00020105 | 2,427458725 | H-DMR |
| chr7 | chr7:1059528-1059877 | Island | 537 | intronic | C7orf50 | 7p22.3 | 0,44614313 | 0,94019799 | 0,00020212 | 1,075457972 | L-DMR |
| chr7 | . | . | 383 | intronic | JAZF1 | 7p15.1 | 0,24812203 | 0,86302888 | 0,00022302 | 1,798359004 | L-DMR |
| chr11 | . | . | 260 | intergenic | GSTP1(dist=6764),NDUFV1(dist=13175) | 11q13.2 | 0,15864376 | 0,85622336 | 0,00027759 | 2,43219642 | H-DMR |
| chr6 | . | . | 208 | intronic | FYN | 6q21 | 0,05257148 | 0,80325993 | 0,00031746 | 3,933514655 | H-DMR |
| chr19 | chr19:2588375-2588590 | Island | 809 | intronic | GNG7 | 19p13.3 | 0,11572332 | 0,7748663 | 0,00034981 | 2,743267782 | H-DMR |
| chr19 | chr19:41035100-41035440 | N_Shelf | 261 | intronic | SPTBN4 | 19q13.2 | 0,08135464 | 0,75837082 | 0,00039473 | 3,220606918 | H-DMR |
| chr5 | . | . | 414 | intronic | ERGIC1 | 5q35.1 | 0,20006195 | 0,87054508 | 0,00044587 | 2,121472202 | H-DMR |
| chr8 | . | . | 363 | intergenic | FBXO32(dist=75379),KLHL38(dist=28680) | 8q24.13 | 0,10520564 | 0,83671033 | 0,00046478 | 2,991516198 | H-DMR |
| chr6 | . | . | 178 | ncRNA_intronic | STL | 6q22.31 | 0,09416539 | 0,88939741 | 0,00060065 | 3,239559396 | H-DMR |
| chr9 | . | . | 321 | intronic | ERCC6L2 | 9q22.32 | 0,16284588 | 0,76150747 | 0,0006422 | 2,225350969 | H-DMR |
| chr3 | chr3:14988612-14989036 | N_Shelf | 202 | ncRNA_exonic | FGD5-AS1 | 3p25.1 | 0,05746943 | 0,82256868 | 0,00070903 | 3,839269496 | H-DMR |
| chr1 | . | . | 195 | ncRNA_intronic | LEMD1-AS1 | 1q32.1 | 0,102218 | 0,82565112 | 0,00077831 | 3,013883029 | H-DMR |
| chr6 | . | . | 538 | ncRNA_intronic | MGC39372 | 6p25.2 | 0,1540822 | 0,72328779 | 0,00126591 | 2,23086959 | H-DMR |
| chr10 | . | . | 204 | intronic | USP6NL | 10p14 | 0,20104981 | 0,8977808 | 0,00139246 | 2,15881027 | H-DMR |
| chr19 | . | . | 522 | intronic | GNG7 | 19p13.3 | 0,12948916 | 0,73889088 | 0,00139901 | 2,512529992 | H-DMR |
| chr17 | . | . | 198 | ncRNA_exonic | LOC100128288 | 17p13.1 | 0,05781477 | 0,79021174 | 0,00141907 | 3,772729269 | H-DMR |
| chr19 | . | . | 278 | intronic | CHST8 | 19q13.11 | 0,08022458 | 0,86378561 | 0,00147303 | 3,428557046 | H-DMR |
| chr2 | . | . | 244 | intronic | NRXN1 | 2p16.3 | 0,15608846 | 0,83038284 | 0,00178647 | 2,411412751 | H-DMR |
| chr3 | . | . | 236 | intronic | FNDC3B | 3q26.31 | 0,26101952 | 0,8517858 | 0,00207306 | 1,706332978 | L-DMR |
| chr4 | . | . | 191 | intronic | RGS12 | 4p16.3 | 0,39079735 | 0,93577252 | 0,00243821 | 1,259737179 | L-DMR |
| chr14 | chr14:103799809-103801661 | N_Shelf | 269 | intergenic | LINC00605(dist=140944),EIF5(dist=3761) | 14q32.32 | 0,0670191 | 0,52759982 | 0,00248073 | 2,976799855 | H-DMR |
| chr7 | . | . | 619 | intronic | HIPK2 | 7q34 | 0,07175424 | 0,72516752 | 0,00248969 | 3,33717832 | H-DMR |
| chr4 | . | . | 203 | intronic | GAB1 | 4q31.21 | 0,08212018 | 0,78925798 | 0,00250825 | 3,264688248 | H-DMR |
| chr7 | . | . | 258 | ncRNA_intronic | FAM133DP | 7q21.2 | 0,26921 | 0,90600361 | 0,00259066 | 1,750784798 | L-DMR |
| chr7 | . | . | 243 | UTR5 | NOS3 | 7q36.1 | 0,15400773 | 0,82044563 | 0,00261319 | 2,413404966 | H-DMR |
| chr10 | chr10:99185778-99186294 | S_Shelf | 318 | intronic | PGAM1 | 10q24.1 | 0,17863506 | 0,74897647 | 0,00283834 | 2,067905135 | H-DMR |
| chr12 | . | . | 268 | intronic | C2CD5 | 12p12.1 | 0,18682343 | 0,86577436 | 0,00288202 | 2,212315677 | H-DMR |
| chr8 | . | . | 339 | intronic | SDC2 | 8q22.1 | 0,14321326 | 0,75311714 | 0,00329775 | 2,394709203 | H-DMR |
| chr11 | . | . | 352 | intronic | TUB | 11p15.4 | 0,12465158 | 0,73467199 | 0,00349634 | 2,559199102 | H-DMR |
| chr2 | . | . | 306 | intronic | HPCAL1 | 2p25.1 | 0,12431771 | 0,79574103 | 0,00384627 | 2,678267155 | H-DMR |
| chr13 | . | . | 590 | intronic | FOXO1 | 13q14.11 | 0,13794705 | 0,74561197 | 0,00385236 | 2,434310416 | H-DMR |
| chr3 | . | . | 251 | intronic | LARS2 | 3p21.31 | 0,2955984 | 0,86072411 | 0,00389266 | 1,541912421 | L-DMR |
| chr3 | . | . | 386 | intergenic | MIR548I1(dist=72159),FAM86JP(dist=53504) | 3q21.2 | 0,16352772 | 0,84412761 | 0,00395509 | 2,367925902 | H-DMR |
| chr11 | . | . | 283 | intergenic | CTSC(dist=19782),GRM5(dist=146738) | 11q14.2 | 0,12126182 | 0,85967644 | 0,00400348 | 2,825668388 | H-DMR |
| chr16 | chr16:1152412-1152700 | N_Shelf | 384 | intergenic | C1QTNF8(dist=3745),CACNA1H(dist=52868) | 16p13.3 | 0,37047793 | 0,86251284 | 0,0042664 | 1,219158333 | L-DMR |
| chr2 | . | . | 216 | intronic | SCTR | 2q14.2 | 0,2286556 | 0,88638404 | 0,00451232 | 1,954755652 | L-DMR |
| chr12 | . | . | 200 | intronic | MED13L | 12q24.21 | 0,30502974 | 0,87016762 | 0,00499501 | 1,512343423 | L-DMR |
| chr4 | . | . | 246 | intronic | SMAD1 | 4q31.21 | 0,10842778 | 0,77377663 | 0,00528527 | 2,835182723 | H-DMR |
| chr17 | . | . | 275 | exonic | SERPINF2 | 17p13.3 | 0,25207321 | 0,85515047 | 0,00538712 | 1,762335496 | L-DMR |
| chr8 | . | . | 194 | intronic | NSMCE2 | 8q24.13 | 0,04262034 | 0,76906854 | 0,00548503 | 4,173498171 | H-DMR |
| chr17 | chr17:79377529-79377870 | Island | 483 | intronic | BAHCC1 | 17q25.3 | 0,2278225 | 0,73468702 | 0,00551378 | 1,68921955 | L-DMR |
| chr20 | . | . | 369 | intergenic | B4GALT5(dist=13727),SLC9A8(dist=84733) | 20q13.13 | 0,23034311 | 0,86904859 | 0,00568999 | 1,915652398 | L-DMR |
| chr5 | chr5:1494853-1495287 | S_Shelf | 313 | intronic | LPCAT1 | 5p15.33 | 0,07195797 | 0,77362073 | 0,00591486 | 3,426400061 | H-DMR |
| chr3 | . | . | 197 | intronic | SEC62 | 3q26.2 | 0,14179115 | 0,78080649 | 0,00629421 | 2,461197556 | H-DMR |
| chr12 | chr12:96389405-96389675 | S_Shore | 668 | UTR5 | HAL | 12q23.1 | 0,12636759 | 0,62072423 | 0,00631494 | 2,296325965 | H-DMR |
| chr2 | chr2:98962873-98964187 | N_Shore | 239 | upstream | CNGA3 | 2q11.2 | 0,08897334 | 0,73778204 | 0,00759309 | 3,051749653 | H-DMR |
| chr12 | chr12:51818460-51819166 | S_Shelf | 242 | intronic | SLC4A8 | 12q13.13 | 0,22217111 | 0,85573962 | 0,00765721 | 1,945500659 | L-DMR |
| chr16 | . | . | 210 | intronic | IL34 | 16q22.1 | 0,02732532 | 0,57732123 | 0,00788566 | 4,401063983 | H-DMR |
| chr19 | chr19:911689-911996 | N_Shelf | 537 | intronic | R3HDM4 | 19p13.3 | 0,25949611 | 0,72381197 | 0,00818054 | 1,479902055 | L-DMR |
| chr7 | chr7:4832111-4832481 | S_Shelf | 305 | intergenic | AP5Z1(dist=2111),RADIL(dist=2298) | 7p22.1 | 0,21351779 | 0,81582434 | 0,00827569 | 1,933902272 | L-DMR |
| chr1 | . | . | 285 | intronic | CSMD2 | 1p35.1 | 0,09242653 | 0,70799027 | 0,00829725 | 2,937350608 | H-DMR |
| chr2 | . | . | 290 | intergenic | MIR4262(dist=36730),AC096559.1(dist=133110) | 2p25.1 | 0,12624458 | 0,69719326 | 0,00829811 | 2,465337172 | H-DMR |
| chr3 | . | . | 166 | intronic | LARS2 | 3p21.31 | 0,07708871 | 0,75168406 | 0,00846066 | 3,285534919 | H-DMR |
| chr1 | . | . | 281 | ncRNA_exonic | KCND3-IT1 | 1p13.2 | 0,08343256 | 0,76105087 | 0,00849679 | 3,189310471 | H-DMR |
| chr2 | . | . | 299 | intronic | HPCAL1 | 2p25.1 | 0,13175622 | 0,81104409 | 0,00872261 | 2,621909274 | H-DMR |
| chr22 | . | . | 282 | intronic | ATXN10 | 22q13.31 | 0,24473294 | 0,92681327 | 0,00980371 | 1,921070406 | L-DMR |
| chr1 | . | . | 304 | intronic | GALNT2 | 1q42.13 | 0,22232954 | 0,82061598 | 0,00998233 | 1,884007602 | L-DMR |
| chr11 | . | . | 310 | intergenic | LINC00900(dist=347129),BUD13(dist=640529) | 11q23.3 | 0,26651614 | 0,81706647 | 0,01007186 | 1,616230545 | L-DMR |
| chr3 | chr3:71773826-71774873 | S_Shore | 531 | intronic | EIF4E3 | 3p13 | 0,34559721 | 0,91571288 | 0,01013593 | 1,405803744 | L-DMR |
| chr12 | . | . | 442 | intergenic | MMP17(dist=9068),ULK1(dist=33453) | 12q24.33 | 0,21433045 | 0,66412119 | 0,01050682 | 1,631609702 | L-DMR |
| chr6 | . | . | 309 | intronic | PARK2 | 6q26 | 0,71848301 | 0,11913041 | 0,01062497 | -2,59241231 | H-DMR |
| chr12 | . | . | 212 | intergenic | NEDD1(dist=352596),RMST(dist=158522) | 12q23.1 | 0,09048026 | 0,76735137 | 0,01097358 | 3,084212359 | H-DMR |
| chr12 | . | . | 200 | intronic | ERP29 | 12q24.13 | 0,12214358 | 0,85555219 | 0,01116253 | 2,808277828 | H-DMR |
| chr8 | . | . | 620 | intergenic | LINC00589(dist=157046),FAM183CP(dist=15738) | 8p12 | 0,19762316 | 0,75300207 | 0,01118488 | 1,929901801 | L-DMR |
| chr5 | . | . | 503 | intronic | EFNA5 | 5q21.3 | 0,06177447 | 0,38464511 | 0,01123309 | 2,638445334 | H-DMR |
| chr3 | . | . | 232 | intronic | CACNA1D | 3p21.1 | 0,22243759 | 0,88375376 | 0,0112401 | 1,990243837 | L-DMR |
| chr2 | . | . | 317 | intronic | NOSTRIN | 2q24.3 | 0,42226248 | 0,90690446 | 0,01173275 | 1,102810512 | L-DMR |
| chr7 | . | . | 209 | intronic | IMMP2L | 7q31.1 | 0,16885328 | 0,85241586 | 0,01191897 | 2,335787232 | H-DMR |
| chr13 | . | . | 265 | ncRNA_intronic | DIAPH3-AS1 | 13q21.2 | 0,23051113 | 0,8013817 | 0,01240033 | 1,797653154 | L-DMR |
| chr8 | . | . | 229 | intronic | WRN | 8p12 | 0,14551127 | 0,81373458 | 0,01270717 | 2,483427404 | H-DMR |
| chr2 | chr2:42795847-42796230 | N_Shore | 250 | intronic | MTA3 | 2p21 | 0,00874126 | 0,76257474 | 0,01285209 | 6,446893682 | H-DMR |
| chr1 | . | . | 189 | intronic | SIPA1L2 | 1q42.2 | 0,26679632 | 0,92654755 | 0,01302423 | 1,796126249 | L-DMR |
| chr12 | . | . | 288 | intergenic | RDH16(dist=28090),GPR182(dist=8559) | 12q13.3 | 0,07593644 | 0,73581301 | 0,01328897 | 3,276474915 | H-DMR |
| chr2 | . | . | 281 | intronic | UPP2 | 2q24.1 | 0,26232524 | 0,82120454 | 0,01331909 | 1,646384978 | L-DMR |
| chr5 | . | . | 485 | intergenic | TNFAIP8(dist=2985),HSD17B4(dist=54369) | 5q23.1 | 0,1424669 | 0,64406947 | 0,01342142 | 2,176589537 | H-DMR |
| chr11 | . | . | 289 | intronic | ME3 | 11q14.2 | 0,52310486 | 0,90047077 | 0,01376752 | 0,783579272 | L-DMR |
| chr8 | chr8:143531118-143534495 | S_Shore | 259 | intergenic | TSNARE1(dist=50266),BAI1(dist=10309) | 8q24.3 | 0,19296571 | 0,53607722 | 0,01389898 | 1,474096327 | L-DMR |
| chr1 | chr1:120254844-120255499 | S_Shore | 512 | intronic | PHGDH | 1p12 | 0,22697121 | 0,8077494 | 0,0141051 | 1,831398462 | L-DMR |
| chr19 | . | . | 543 | intronic | RHPN2 | 19q13.11 | 0,10336801 | 0,68822 | 0,01471072 | 2,735080044 | H-DMR |
| chr10 | . | . | 443 | intronic | ZMIZ1 | 10q22.3 | 0,13938154 | 0,71042639 | 0,0147651 | 2,349645674 | H-DMR |
| chr8 | . | . | 204 | intronic | SPAG1 | 8q22.2 | 0,17549628 | 0,76008427 | 0,01494491 | 2,114718928 | H-DMR |
| chr2 | . | . | 203 | intergenic | C2orf48(dist=14483),HPCAL1(dist=77402) | 2p25.1 | 0,19550147 | 0,82818188 | 0,01499309 | 2,082768183 | H-DMR |
| chr7 | . | . | 606 | intronic | ELMO1 | 7p14.1 | 0,09818948 | 0,6556273 | 0,01524962 | 2,739235561 | H-DMR |
| chr7 | . | . | 178 | intergenic | SMKR1(dist=37896),NRF1(dist=60708) | 7q32.1 | 0,05146833 | 0,70725821 | 0,01539133 | 3,780480142 | H-DMR |
| chr21 | . | . | 268 | intronic | AGPAT3 | 21q22.3 | 0,17529991 | 0,96655206 | 0,01541529 | 2,463022185 | H-DMR |
| chr3 | . | . | 318 | intronic | SLC12A8 | 3q21.2 | 0,16636196 | 0,87309677 | 0,01570174 | 2,391815977 | H-DMR |
| chr16 | . | . | 334 | intronic | ZNRF1 | 16q23.1 | 0,06845963 | 0,74071837 | 0,01704129 | 3,435599717 | H-DMR |
| chr12 | . | . | 670 | intergenic | LINC00936(dist=128159),LINC00615(dist=1077242) | 12q21.33 | 0,18074927 | 0,79834195 | 0,01754671 | 2,143017001 | H-DMR |
| chr5 | chr5:153784641-153785130 | S_Shore | 132 | ncRNA_intronic | SAP30L-AS1 | 5q33.2 | 0,17803228 | 0,90634664 | 0,01769979 | 2,347924079 | H-DMR |
| chr11 | chr11:68178918-68179162 | N_Shelf | 244 | intronic | LRP5 | 11q13.2 | 0,32628296 | 0,89484925 | 0,01792228 | 1,455521015 | L-DMR |
| chr21 | . | . | 106 | ncRNA_intronic | LINC00310 | 21q22.11 | 0,42054064 | 0,95948378 | 0,01809049 | 1,190013192 | L-DMR |
| chr3 | . | . | 162 | intronic | ARHGEF3 | 3p14.3 | 0,00802139 | 0,67418524 | 0,01865632 | 6,393148974 | H-DMR |
| chr2 | chr2:38829103-38830804 | N_Shore | 157 | intronic | HNRNPLL | 2p22.1 | 0,03065292 | 0,42219513 | 0,01880757 | 3,783813521 | H-DMR |
| chr7 | . | . | 173 | intronic | PTPRN2 | 7q36.3 | 0,05479725 | 0,66060796 | 0,01936415 | 3,591618955 | H-DMR |
| chr7 | . | . | 433 | intronic | TTYH3 | 7p22.3 | 0,05823675 | 0,63866139 | 0,01985027 | 3,455049485 | H-DMR |
| chr1 | chr1:42611485-42611691 | N_Shelf | 245 | intergenic | NONE(dist=NONE),GUCA2B(dist=9836) | 1p34.2 | 0,0963913 | 0,77495103 | 0,01999946 | 3,007130304 | H-DMR |
| chr11 | . | . | 252 | intronic | LDLRAD3 | 11p13 | 0,81749477 | 0,23561214 | 0,0205542 | -1,794795624 | L-DMR |
| chr20 | . | . | 229 | exonic | ASXL1 | 20q11.21 | 0,25057627 | 0,9001418 | 0,02061067 | 1,844902493 | L-DMR |
| chr12 | . | . | 373 | intergenic | PITPNM2(dist=21631),MPHOSPH9(dist=23964) | 12q24.31 | 0,16990216 | 0,80830562 | 0,02063938 | 2,250196685 | H-DMR |
| chr22 | chr22:26879458-26879930 | N_Shelf | 763 | exonic | HPS4 | 22q12.1 | 0,11519696 | 0,53886083 | 0,02067617 | 2,225810075 | H-DMR |
| chr7 | . | . | 239 | intronic | MAD1L1 | 7p22.3 | 0,14866947 | 0,78172292 | 0,02113127 | 2,394548925 | H-DMR |
| chr10 | . | . | 398 | intronic | VTI1A | 10q25.2 | 0,13678262 | 0,77429628 | 0,02171835 | 2,501000782 | H-DMR |
| chr18 | . | . | 725 | intergenic | SMAD7(dist=47002),DYM(dist=45364) | 18q21.1 | 0,53941934 | 0,89414379 | 0,02185743 | 0,729099606 | L-DMR |
| chr16 | . | . | 295 | intronic | ITFG3 | 16p13.3 | 0,09225082 | 0,78866582 | 0,02203535 | 3,095780479 | H-DMR |
| chr20 | . | . | 236 | intergenic | ZMYND8(dist=107763),NCOA3(dist=36969) | 20q13.12 | 0,06189533 | 0,49155945 | 0,02223104 | 2,989463442 | H-DMR |
| chr17 | . | . | 201 | intronic | ABR | 17p13.3 | 0,29466204 | 0,9379198 | 0,02255168 | 1,670403349 | L-DMR |
| chr3 | . | . | 238 | intronic | FOXP1 | 3p13 | 0,16454822 | 0,81792357 | 0,02300036 | 2,313455618 | H-DMR |
| chr5 | . | . | 343 | intronic | SLC25A46 | 5q22.1 | 0,12107847 | 0,70025565 | 0,02305524 | 2,531939369 | H-DMR |
| chr9 | . | . | 194 | intergenic | MIR147A(dist=99333),CDK5RAP2(dist=44292) | 9q33.2 | 0,30459588 | 0,87630316 | 0,02328345 | 1,524533633 | L-DMR |
| chr22 | chr22:19278671-19279573 | S_Shore | 541 | upstream | CLTCL1 | 22q11.21 | 0,11179357 | 0,56674816 | 0,02340461 | 2,341870591 | H-DMR |
| chr6 | . | . | 340 | intergenic | ZC3H12D(dist=14141),PPIL4(dist=5002) | 6q25.1 | 0,2175573 | 0,84212541 | 0,02379541 | 1,95263967 | L-DMR |
| chr1 | . | . | 181 | intronic | PBX1 | 1q23.3 | 0,2132933 | 0,85278344 | 0,02442593 | 1,999340775 | L-DMR |
| chr5 | . | . | 232 | intergenic | TSPAN17(dist=36048),LOC102577424(dist=47867) | 5q35.2 | 0,16610825 | 0,7452067 | 0,0250458 | 2,165516917 | H-DMR |
| chr3 | . | . | 177 | ncRNA_intronic | LINC00880 | 3q25.31 | 0,03991571 | 0,84201115 | 0,02743217 | 4,398810759 | H-DMR |
| chr6 | . | . | 283 | intronic | JARID2 | 6p22.3 | 0,13549966 | 0,84271433 | 0,02743844 | 2,636754426 | H-DMR |
| chr1 | . | . | 128 | ncRNA_exonic | MROH7-TTC4 | 1p32.3 | 0,51964692 | 0,90554135 | 0,02848074 | 0,80124882 | L-DMR |
| chr1 | . | . | 190 | exonic | KIAA0040 | 1q25.1 | 0,25634637 | 0,91181818 | 0,02865309 | 1,830651706 | L-DMR |
| chr2 | . | . | 316 | intergenic | ST3GAL5(dist=35734),LOC90784(dist=95132) | 2p11.2 | 0,23700668 | 0,71996962 | 0,02888336 | 1,60300831 | L-DMR |
| chr5 | . | . | 30 | downstream | FAM153A | 5q35.3 | 0,0569072 | 0,68681041 | 0,02958502 | 3,593228805 | H-DMR |
| chr17 | . | . | 308 | exonic | ERN1 | 17q23.3 | 0,25109858 | 0,8272044 | 0,02989488 | 1,71998999 | L-DMR |
| chr17 | . | . | 522 | intronic | RPTOR | 17q25.3 | 0,29587114 | 0,85656441 | 0,03040047 | 1,533592756 | L-DMR |
| chr1 | . | . | 406 | intronic | KCND3 | 1p13.2 | 0,35630185 | 0,87258334 | 0,0318103 | 1,292192955 | L-DMR |
| chr20 | . | . | 335 | intergenic | RP11-347D21.3(dist=293806),LINC00494(dist=75652) | 20q13.13 | 0,23001369 | 0,79755981 | 0,031921 | 1,793872982 | L-DMR |
| chr20 | . | . | 300 | intronic | MACROD2 | 20p12.1 | 0,20617972 | 0,84442594 | 0,03216949 | 2,034068463 | H-DMR |
| chr15 | . | . | 523 | intronic | ISG20 | 15q26.1 | 0,33181074 | 0,81845881 | 0,03275744 | 1,302549229 | L-DMR |
| chr7 | . | . | 294 | intronic | PHTF2 | 7q21.11 | 0,26605837 | 0,89518705 | 0,03280018 | 1,750446376 | L-DMR |
| chr13 | . | . | 246 | intergenic | ABHD13(dist=34090),TNFSF13B(dist=1038) | 13q33.3 | 0,5884497 | 0,92187503 | 0,03385709 | 0,647652091 | L-DMR |
| chr12 | . | . | 302 | exonic | LTBR | 12p13.31 | 0,16948005 | 0,81488829 | 0,03418779 | 2,26548684 | H-DMR |
| chr11 | . | . | 178 | intergenic | SLN(dist=65184),SLC35F2(dist=13568) | 11q22.3 | 0,1234261 | 0,71288749 | 0,03433084 | 2,530026902 | H-DMR |
| chr2 | . | . | 489 | intronic | HDAC4 | 2q37.3 | 0,2495144 | 0,64841275 | 0,03440795 | 1,377789381 | L-DMR |
| chr8 | . | . | 235 | exonic | SPIDR | 8q11.21 | 0,31014306 | 0,89999244 | 0,03505583 | 1,53697904 | L-DMR |
| chr2 | . | . | 218 | intronic | SLC35F5 | 2q14.1 | 0,13152147 | 0,61177979 | 0,03530792 | 2,21771412 | H-DMR |
| chr5 | . | . | 285 | exonic | MARCH6 | 5p15.2 | 0,25586313 | 0,91046911 | 0,03537966 | 1,831237802 | L-DMR |
| chr12 | . | . | 213 | intronic | SYT1 | 12q21.2 | 0,18354399 | 0,75533289 | 0,03625443 | 2,040986733 | H-DMR |
| chr2 | . | . | 298 | intergenic | NCK2(dist=16621),C2orf40(dist=154464) | 2q12.2 | 0,25683518 | 0,87102176 | 0,03642035 | 1,761865932 | L-DMR |
| chr1 | . | . | 351 | intronic | LPAR3 | 1p22.3 | 0,11903205 | 0,53413762 | 0,03678441 | 2,16586142 | H-DMR |
| chr22 | . | . | 300 | intronic | TCF20 | 22q13.2 | 0,16571805 | 0,8956053 | 0,03687001 | 2,434132316 | H-DMR |
| chr17 | . | . | 370 | intergenic | HELZ(dist=89673),PSMD12(dist=5257) | 17q24.2 | 0,35660315 | 0,79671266 | 0,03712338 | 1,159740053 | L-DMR |
| chr8 | . | . | 284 | intronic | TRAPPC9 | 8q24.3 | 0,1316135 | 0,81852984 | 0,03728612 | 2,636727534 | H-DMR |
| chr17 | . | . | 326 | ncRNA_intronic | RAD51L3-RFFL | 17q12 | 0,08661986 | 0,79500023 | 0,03728676 | 3,198185532 | H-DMR |
| chr2 | . | . | 312 | intronic | CERS6 | 2q24.3 | 0,37173631 | 0,91121063 | 0,03802997 | 1,293504964 | L-DMR |
| chr10 | . | . | 413 | intronic | ANK3 | 10q21.2 | 0,36554624 | 0,76622517 | 0,03833923 | 1,067714506 | L-DMR |
| chr4 | . | . | 348 | intronic | COPS4 | 4q21.22 | 0,13367199 | 0,7974605 | 0,03885295 | 2,57671587 | H-DMR |
| chr12 | chr12:50349079-50349525 | N_Shore | 290 | intronic | AQP2 | 12q13.12 | 0,26322409 | 0,79761982 | 0,03905907 | 1,599409731 | L-DMR |
| chr2 | . | . | 499 | intergenic | WDR43(dist=3680),FAM179A(dist=28905) | 2p23.2 | 0,33463215 | 0,78033402 | 0,03906913 | 1,221515738 | L-DMR |
| chr6 | . | . | 194 | intronic | TFEB | 6p21.1 | 0,17940444 | 0,77598176 | 0,03972079 | 2,112807146 | H-DMR |
| chr3 | . | . | 498 | intergenic | DNASE1L3(dist=3592),ABHD6(dist=22439) | 3p14.3 | 0,34605772 | 0,88099736 | 0,04151001 | 1,348125007 | L-DMR |
| chr5 | chr5:293625-294130 | S_Shelf | 470 | intronic | PDCD6 | 5p15.33 | 0,63170745 | 0,8729012 | 0,04189251 | 0,466561784 | L-DMR |
| chr7 | . | . | 235 | intronic | IMMP2L | 7q31.1 | 0,44684584 | 0,85235394 | 0,04198113 | 0,931675441 | L-DMR |
| chr10 | . | . | 293 | intronic | VCL | 10q22.2 | 0,30266255 | 0,90095667 | 0,04353688 | 1,57374755 | L-DMR |
| chr7 | . | . | 300 | intronic | MAD1L1 | 7p22.3 | 0,17296678 | 0,91606107 | 0,04354474 | 2,4049488 | H-DMR |
| chr9 | . | . | 511 | intergenic | ABCA1(dist=32880),SLC44A1(dist=282976) | 9q31.1 | 0,44602592 | 0,86630802 | 0,04367732 | 0,957752521 | L-DMR |
| chr19 | chr19:56652191-56652684 | N_Shelf | 210 | intergenic | ZNF787(dist=16905),ZNF444(dist=2771) | 19q13.43 | 0,07979798 | 0,80110965 | 0,04380032 | 3,32757559 | H-DMR |
| chr13 | . | . | 890 | intergenic | MYO16(dist=443696),LINC00676(dist=75680) | 13q34 | 0,29853332 | 0,75879663 | 0,04437372 | 1,345821308 | L-DMR |
| chr17 | . | . | 222 | intergenic | NR1D1(dist=6961),MSL1(dist=14629) | 17q21.1 | 0,15724594 | 0,8156294 | 0,0464034 | 2,374891011 | H-DMR |
| chr7 | . | . | 321 | intronic | CUX1 | 7q22.1 | 0,17075335 | 0,85969046 | 0,0464998 | 2,331903414 | H-DMR |
| chr18 | . | . | 280 | exonic | MPPE1 | 18p11.21 | 0,20700136 | 0,81327966 | 0,0467013 | 1,974111286 | L-DMR |
| chr4 | chr4:90228714-90229010 | N_Shore | 215 | intronic | GPRIN3 | 4q22.1 | 0,0602602 | 0,67182725 | 0,04677574 | 3,478812948 | H-DMR |
| chr9 | . | . | 494 | intronic | RALGDS | 9q34.2 | 0,20904666 | 0,73352008 | 0,04679465 | 1,811011467 | L-DMR |
| chr1 | . | . | 151 | intergenic | RP5-855F14.1(dist=53981),IRF2BP2(dist=18358) | 1q42.3 | 0,11677273 | 0,82051623 | 0,04691666 | 2,812828471 | H-DMR |
| chr4 | . | . | 209 | intronic | ARHGAP24 | 4q21.23 | 0,10458027 | 0,77168467 | 0,04709073 | 2,883400746 | H-DMR |
| chr12 | . | . | 169 | intergenic | FAM109A(dist=17939),SH2B3(dist=18719) | 12q24.12 | 0,01162665 | 0,52177891 | 0,04709184 | 5,487931256 | H-DMR |
| chr1 | chr1:23763154-23763971 | S_Shelf | 250 | intronic | ASAP3 | 1p36.12 | 0,13038997 | 0,54160815 | 0,04766748 | 2,054416551 | H-DMR |
| chr1 | . | . | 183 | intronic | RFWD2 | 1q25.2 | 0,18606677 | 0,92341449 | 0,04815645 | 2,311157946 | H-DMR |
| chr17 | chr17:47840834-47841377 | N_Shelf | 231 | intronic | FAM117A | 17q21.33 | 0,20554844 | 0,87825397 | 0,0484783 | 2,09515977 | H-DMR |
| chr7 | . | . | 414 | intronic | POR | 7q11.23 | 0,07327902 | 0,67460179 | 0,04978379 | 3,202564032 | H-DMR |
