# Supplementary material for: Methylome Analysis in Nonfunctioning and GH-Secreting Pituitary Adenomas
Source: Front Endocrinol (Lausanne). 2022 Mar 30;13:841118. doi: 10.3389/fendo.2022.841118 (PMC9007725; doi:10.3389/fendo.2022.841118)
Supplement: Supplementary file 3 [file Table_2.docx]

**Supplementary Table 2. Gene Ontology Biological Process Enrichment of DMR-related genes.**

| **Biological Process** | **GO term ID** | **Enrichment FDR** | **Query Genes** | **Gene Set Size** | **Fold Enrichment** |
| --- | --- | --- | --- | --- | --- |
| Cell development | GO:0048468 | 0,000190157 | 41 | 2194 | 2,448254906 |
| Central nervous system development | GO:0007417 | 0,005409151 | 24 | 1107 | 2,840357599 |
| Regulation of developmental process | GO:0050793 | 0,005409151 | 42 | 2693 | 2,043253902 |
| Regulation of cell communication | GO:0010646 | 0,006560495 | 52 | 3777 | 1,803706037 |
| Plasma membrane bounded cell projection organization | GO:0120036 | 0,006560495 | 30 | 1650 | 2,382027168 |
| Cell projection organization | GO:0030030 | 0,007769785 | 30 | 1691 | 2,324272518 |
| Nervous system development | GO:0007399 | 0,008363897 | 39 | 2583 | 1,978106185 |
| Response to endogenous stimulus | GO:0009719 | 0,008363897 | 30 | 1768 | 2,223045717 |
| Vesicle-mediated transport | GO:0016192 | 0,008363897 | 36 | 2318 | 2,034691024 |
| Neurogenesis | GO:0022008 | 0,008363897 | 30 | 1757 | 2,236963476 |
| Regulation of signaling | GO:0023051 | 0,008363897 | 51 | 3815 | 1,751398744 |
| Regulation of localization | GO:0032879 | 0,008363897 | 43 | 2951 | 1,909011946 |
| Generation of neurons | GO:0048699 | 0,008363897 | 29 | 1624 | 2,339490969 |
| Endoplasmic reticulum to Golgi vesicle-mediated transport | GO:0006888 | 0,009241153 | 9 | 218 | 5,408731414 |
| Anatomical structure morphogenesis | GO:0009653 | 0,009241153 | 41 | 2871 | 1,870940879 |
| Neuron projection development | GO:0031175 | 0,009241153 | 21 | 1045 | 2,63276687 |
| Muscle cell proliferation | GO:0033002 | 0,009241153 | 9 | 215 | 5,484202085 |
| Intracellular signal transduction | GO:0035556 | 0,009241153 | 42 | 2975 | 1,849574037 |
| Positive regulation of molecular function | GO:0044093 | 0,009241153 | 31 | 1916 | 2,119705805 |
| Neuron development | GO:0048666 | 0,009241153 | 23 | 1203 | 2,504791661 |
| Cellular developmental process | GO:0048869 | 0,009241153 | 59 | 4738 | 1,631422153 |
| Neuron differentiation | GO:0030182 | 0,009502406 | 26 | 1473 | 2,312490734 |
| Regulation of catalytic activity | GO:0050790 | 0,009510102 | 39 | 2697 | 1,894493243 |
| Regulation of molecular function | GO:0065009 | 0,010229416 | 46 | 3424 | 1,760084327 |
| Neuron projection morphogenesis | GO:0048812 | 0,011782389 | 16 | 686 | 3,055661674 |
| Cell differentiation | GO:0030154 | 0,012263252 | 57 | 4645 | 1,607676033 |
| Cellular response to inorganic substance | GO:0071241 | 0,012379171 | 9 | 236 | 4,996201052 |
| Regulation of signal transduction | GO:0009966 | 0,012427638 | 45 | 3379 | 1,744752069 |
| Positive regulation of cellular metabolic process | GO:0031325 | 0,012427638 | 46 | 3490 | 1,726799065 |
| Homeostatic process | GO:0042592 | 0,012427638 | 32 | 2078 | 2,017501355 |
| Cell projection morphogenesis | GO:0048858 | 0,012427638 | 16 | 704 | 2,97753396 |
| Plasma membrane bounded cell projection morphogenesis | GO:0120039 | 0,012427638 | 16 | 700 | 2,99454844 |
| Positive regulation of nucleobase-containing compound metabolic process | GO:0045935 | 0,014756406 | 31 | 2014 | 2,016562225 |
| Cell part morphogenesis | GO:0032990 | 0,015378512 | 16 | 721 | 2,907328583 |
| Positive regulation of smooth muscle cell proliferation | GO:0048661 | 0,016235481 | 6 | 104 | 7,558355438 |
| Regulation of biological quality | GO:0065008 | 0,016884078 | 54 | 4420 | 1,600592916 |
| Response to inorganic substance | GO:0010035 | 0,017046485 | 14 | 584 | 3,140686506 |
| Cellular component morphogenesis | GO:0032989 | 0,01772139 | 17 | 815 | 2,732755095 |
| Cellular response to endogenous stimulus | GO:0071495 | 0,019151564 | 25 | 1503 | 2,179166571 |
| Regulation of mitotic nuclear division | GO:0007088 | 0,019311819 | 6 | 110 | 7,146081505 |
| Regulation of smooth muscle cell proliferation | GO:0048660 | 0,019488917 | 7 | 157 | 5,841276814 |
| Movement of cell or subcellular component | GO:0006928 | 0,019564688 | 33 | 2271 | 1,903733734 |
| Positive regulation of nitrogen compound metabolic process | GO:0051173 | 0,019564688 | 43 | 3287 | 1,713871084 |
| Cell morphogenesis | GO:0000902 | 0,019795675 | 20 | 1085 | 2,414958419 |
| Response to hormone | GO:0009725 | 0,019795675 | 19 | 998 | 2,494206804 |
| Regulation of system process | GO:0044057 | 0,019795675 | 14 | 606 | 3,026668184 |
| Smooth muscle cell proliferation | GO:0048659 | 0,019795675 | 7 | 160 | 5,731752874 |
| Organelle organization | GO:0006996 | 0,020880192 | 53 | 4394 | 1,580247882 |
| Negative regulation of cytoskeleton organization | GO:0051494 | 0,022461694 | 7 | 166 | 5,524581083 |
| Bone development | GO:0060348 | 0,022461694 | 8 | 219 | 4,785808009 |
| Positive regulation of metabolic process | GO:0009893 | 0,022690991 | 50 | 4101 | 1,597311561 |
| Peptidyl-amino acid modification | GO:0018193 | 0,022690991 | 23 | 1368 | 2,202678631 |
| Positive regulation of mitotic nuclear division | GO:0045840 | 0,022690991 | 4 | 43 | 12,18711574 |
| Positive regulation of developmental process | GO:0051094 | 0,022690991 | 23 | 1373 | 2,194657223 |
| Protein depolymerization | GO:0051261 | 0,022690991 | 6 | 120 | 6,550574713 |
| Regulation of intracellular signal transduction | GO:1902531 | 0,024167688 | 29 | 1940 | 1,958419244 |
| Circulatory system process | GO:0003013 | 0,024381291 | 14 | 632 | 2,902153354 |
| Response to magnesium ion | GO:0032026 | 0,024381291 | 3 | 19 | 20,68602541 |
| Negative regulation of protein polymerization | GO:0032272 | 0,024381291 | 5 | 83 | 7,89225869 |
| Cellular response to hormone stimulus | GO:0032870 | 0,024381291 | 15 | 715 | 2,748492886 |
| Regulation of organelle organization | GO:0033043 | 0,024381291 | 22 | 1309 | 2,201873853 |
| Regulation of muscle adaptation | GO:0043502 | 0,024381291 | 5 | 83 | 7,89225869 |
| Regulation of cellular component biogenesis | GO:0044087 | 0,024381291 | 19 | 1044 | 2,384308803 |
| Developmental growth | GO:0048589 | 0,024381291 | 14 | 640 | 2,865876437 |
| Positive regulation of developmental growth | GO:0048639 | 0,024381291 | 7 | 174 | 5,270577355 |
| Regulation of cellular component organization | GO:0051128 | 0,024381291 | 35 | 2555 | 1,794678003 |
| Actin filament capping | GO:0051693 | 0,024381291 | 4 | 45 | 11,64546616 |
| Dendritic spine maintenance | GO:0097062 | 0,024381291 | 3 | 19 | 20,68602541 |
| Cellular response to oxygen-containing compound | GO:1901701 | 0,024381291 | 22 | 1312 | 2,19683908 |
| Cell migration | GO:0016477 | 0,024843768 | 25 | 1590 | 2,059929155 |
| Brain development | GO:0007420 | 0,02683201 | 16 | 811 | 2,584690392 |
| Locomotion | GO:0040011 | 0,02683201 | 29 | 1982 | 1,916918937 |
| Negative regulation of actin filament depolymerization | GO:0030835 | 0,027514573 | 4 | 49 | 10,69481586 |
| Thymus development | GO:0048538 | 0,027514573 | 4 | 49 | 10,69481586 |
| Positive regulation of mitotic cell cycle phase transition | GO:1901992 | 0,027855161 | 5 | 87 | 7,529396221 |
| Regulation of mitotic cell cycle | GO:0007346 | 0,02791525 | 14 | 661 | 2,774827412 |
| Vesicle organization | GO:0016050 | 0,02791525 | 10 | 370 | 3,540851196 |
| Negative regulation of filopodium assembly | GO:0051490 | 0,02791525 | 2 | 5 | 52,4045977 |
| SMAD protein signal transduction | GO:0060395 | 0,02791525 | 5 | 88 | 7,443834901 |
| Positive regulation of cyclase activity | GO:0031281 | 0,028262366 | 3 | 22 | 17,86520376 |
| Regulation of protein-containing complex disassembly | GO:0043244 | 0,028262366 | 6 | 135 | 5,822733078 |
| Establishment of localization in cell | GO:0051649 | 0,028262366 | 39 | 3018 | 1,692991476 |
| Regulation of cell population proliferation | GO:0042127 | 0,029043703 | 27 | 1817 | 1,946786101 |
| Regulation of protein targeting | GO:1903533 | 0,029043703 | 5 | 90 | 7,278416347 |
| Muscle system process | GO:0003012 | 0,030803984 | 11 | 448 | 3,216800082 |
| Positive regulation of lyase activity | GO:0051349 | 0,030816791 | 3 | 23 | 17,08845577 |
| Positive regulation of cellular component organization | GO:0051130 | 0,030838478 | 21 | 1269 | 2,168038912 |
| Cellular response to organic substance | GO:0071310 | 0,030838478 | 39 | 3045 | 1,677979729 |
| Cellular localization | GO:0051641 | 0,031126556 | 46 | 3803 | 1,584677553 |
| Regulation of nuclear division | GO:0051783 | 0,031126556 | 6 | 140 | 5,614778325 |
| Regulation of protein depolymerization | GO:1901879 | 0,031126556 | 5 | 93 | 7,043628723 |
| MAPK cascade | GO:0000165 | 0,031402851 | 18 | 1015 | 2,323356548 |
| Skeletal system development | GO:0001501 | 0,031402851 | 12 | 527 | 2,98318393 |
| Negative regulation of protein-containing complex disassembly | GO:0043242 | 0,031402851 | 5 | 94 | 6,968696503 |
| Positive regulation of neurotransmitter transport | GO:0051590 | 0,031402851 | 3 | 24 | 16,37643678 |
| Membrane organization | GO:0061024 | 0,031402851 | 18 | 1014 | 2,325647827 |
| Regulation of response to stimulus | GO:0048583 | 0,031404237 | 54 | 4708 | 1,50268069 |
| Regulation of protein phosphorylation | GO:0001932 | 0,032834595 | 21 | 1287 | 2,137716689 |
| Voluntary musculoskeletal movement | GO:0050882 | 0,032954307 | 2 | 6 | 43,67049808 |
| Negative regulation of protein-containing complex assembly | GO:0031333 | 0,033344794 | 6 | 145 | 5,421165279 |
| Regulation of multicellular organismal process | GO:0051239 | 0,033344794 | 38 | 2973 | 1,674549876 |
| Head development | GO:0060322 | 0,033660747 | 16 | 856 | 2,448812977 |
| Enzyme linked receptor protein signaling pathway | GO:0007167 | 0,035141913 | 19 | 1122 | 2,218554716 |
| Regulation of anatomical structure morphogenesis | GO:0022603 | 0,035141913 | 18 | 1034 | 2,28066431 |
| Regulation of actin filament depolymerization | GO:0030834 | 0,035141913 | 4 | 58 | 9,035275466 |
| Regulation of phosphorylation | GO:0042325 | 0,035141913 | 23 | 1486 | 2,027768754 |
| Cell morphogenesis involved in neuron differentiation | GO:0048667 | 0,035141913 | 13 | 620 | 2,747015202 |
| Cell motility | GO:0048870 | 0,035141913 | 26 | 1776 | 1,917961065 |
| Localization of cell | GO:0051674 | 0,035141913 | 26 | 1776 | 1,917961065 |
| Positive regulation of nuclear division | GO:0051785 | 0,035141913 | 4 | 58 | 9,035275466 |
| Cellular response to metal ion | GO:0071248 | 0,037522932 | 7 | 207 | 4,430340385 |
| Positive regulation of growth | GO:0045927 | 0,039802405 | 8 | 272 | 3,853279243 |
| Spontaneous neurotransmitter secretion | GO:0061669 | 0,039802405 | 2 | 7 | 37,4318555 |
| Cellular response to magnesium ion | GO:0071286 | 0,039802405 | 2 | 7 | 37,4318555 |
| Positive regulation of synaptic vesicle exocytosis | GO:2000302 | 0,039802405 | 2 | 7 | 37,4318555 |
| Regulation of mitotic cell cycle phase transition | GO:1901990 | 0,040116657 | 11 | 481 | 2,996104858 |
| Animal organ development | GO:0048513 | 0,040498886 | 46 | 3896 | 1,546850291 |
| Axonogenesis | GO:0007409 | 0,041840901 | 11 | 490 | 2,941074361 |
| Lipid biosynthetic process | GO:0008610 | 0,041840901 | 15 | 805 | 2,441207967 |
| Positive regulation of biosynthetic process | GO:0009891 | 0,041840901 | 29 | 2121 | 1,791293415 |
| Response to organic substance | GO:0010033 | 0,041840901 | 44 | 3700 | 1,557974526 |
| Positive regulation of cell communication | GO:0010647 | 0,041840901 | 27 | 1907 | 1,854908414 |
| Peptidyl-serine modification | GO:0018209 | 0,041840901 | 9 | 347 | 3,397992646 |
| Regulation of phosphate metabolic process | GO:0019220 | 0,041840901 | 25 | 1719 | 1,905344594 |
| Actin filament depolymerization | GO:0030042 | 0,041840901 | 4 | 63 | 8,318190111 |
| Regulation of actin filament bundle assembly | GO:0032231 | 0,041840901 | 5 | 106 | 6,179787465 |
| Positive regulation of catalytic activity | GO:0043085 | 0,041840901 | 23 | 1527 | 1,973323096 |
| Muscle adaptation | GO:0043500 | 0,041840901 | 5 | 106 | 6,179787465 |
| Regulation of phosphorus metabolic process | GO:0051174 | 0,041840901 | 25 | 1719 | 1,905344594 |
| Regulation of protein metabolic process | GO:0051246 | 0,041840901 | 36 | 2841 | 1,660124531 |
| Cellular response to chemical stimulus | GO:0070887 | 0,041840901 | 44 | 3697 | 1,559238774 |
| Plasma membrane bounded cell projection assembly | GO:0120031 | 0,041840901 | 13 | 648 | 2,628317014 |
| Positive regulation of cell cycle phase transition | GO:1901989 | 0,041840901 | 5 | 106 | 6,179787465 |
| Regulation of multicellular organismal development | GO:2000026 | 0,041840901 | 22 | 1438 | 2,004348313 |
| Growth | GO:0040007 | 0,041873654 | 17 | 985 | 2,261112084 |
| Regulation of homotypic cell-cell adhesion | GO:0034110 | 0,04264387 | 3 | 30 | 13,10114943 |
| Barbed-end actin filament capping | GO:0051016 | 0,04264387 | 3 | 30 | 13,10114943 |
| Negative regulation of BMP signaling pathway | GO:0030514 | 0,042758886 | 4 | 65 | 8,0622458 |
| Positive regulation of RNA metabolic process | GO:0051254 | 0,042758886 | 26 | 1832 | 1,859333434 |
| Trans-synaptic signaling by lipid | GO:0099541 | 0,042758886 | 2 | 8 | 32,75287356 |
| Trans-synaptic signaling by endocannabinoid | GO:0099542 | 0,042758886 | 2 | 8 | 32,75287356 |
| Regulation of cellular protein metabolic process | GO:0032268 | 0,043372299 | 34 | 2650 | 1,68090219 |
| Chemical homeostasis | GO:0048878 | 0,043372299 | 20 | 1264 | 2,072966681 |
| Negative regulation of plasma membrane bounded cell projection assembly | GO:0120033 | 0,04466743 | 3 | 31 | 12,6785317 |
| Stem cell population maintenance | GO:0019827 | 0,046433919 | 6 | 167 | 4,706999794 |
| Cell projection assembly | GO:0030031 | 0,046433919 | 13 | 663 | 2,568852828 |
| Negative regulation of actin filament polymerization | GO:0030837 | 0,046433919 | 4 | 68 | 7,706558485 |
| Regulation of protein modification process | GO:0031399 | 0,046433919 | 25 | 1755 | 1,866260602 |
| Actin filament bundle assembly | GO:0051017 | 0,046433919 | 6 | 167 | 4,706999794 |
| Regulation of cellular component movement | GO:0051270 | 0,046433919 | 18 | 1092 | 2,159530125 |
| Positive regulation of cartilage development | GO:0061036 | 0,046433919 | 3 | 32 | 12,28232759 |
| Negative regulation of cellular response to growth factor stimulus | GO:0090288 | 0,046433919 | 5 | 113 | 5,796968772 |
| Tube development | GO:0035295 | 0,046481638 | 18 | 1096 | 2,151648628 |
| Regulation of cell motility | GO:2000145 | 0,047823046 | 17 | 1010 | 2,205143963 |
| Regulation of actin filament organization | GO:0110053 | 0,048174908 | 8 | 294 | 3,564938619 |
| Positive regulation of guanylate cyclase activity | GO:0031284 | 0,048253186 | 2 | 9 | 29,11366539 |
| Bone marrow development | GO:0048539 | 0,048253186 | 2 | 9 | 29,11366539 |
| Positive regulation of protein targeting to membrane | GO:0090314 | 0,048253186 | 3 | 33 | 11,91013584 |
| Maintenance of cell number | GO:0098727 | 0,048253186 | 6 | 170 | 4,623935091 |
| Negative regulation of supramolecular fiber organization | GO:1902904 | 0,048253186 | 6 | 170 | 4,623935091 |
| Response to reactive oxygen species | GO:0000302 | 0,048328815 | 7 | 231 | 3,97004528 |
| Actin filament bundle organization | GO:0061572 | 0,048328815 | 6 | 171 | 4,596894535 |
| Cellular response to external stimulus | GO:0071496 | 0,048539292 | 9 | 366 | 3,221594121 |
